# In this month’s *Bulletin*

**DOI:** 10.2471/BLT.16.000416

**Published:** 2016-04-01

In the editorial section, Vasee S Moorthy et al. (234) present operating principles for health-data platforms. Viroj Tangcharoensathien et al. (235) introduce an upcoming theme issue on vulnerable populations. Sophie Delaunay et al. (236) discuss improved access to information during public health emergencies.

Andréia Azevedo Soares reports on the effects of Mexico’s soda tax (239–240). Fiona Fleck interviews KM Venkat Narayan about the increased prevalence of type 2 diabetes (241–242). 

## Burundi, Ethiopia, Malawi, Rwanda, South Africa, Uganda

### Getting research questions answered

Eva A Rehfuess et al. (297–305) describe a priority-setting approach.

## China, Ghana, India, Mexico, the Russian Federation, South Africa

### Gaps in universal coverage

Christine Goeppel et al. (276–285) document the extent of health coverage for adults with chronic illness.

## India 

### Tracking health by the household

Rakhi Dandona et al. (286–296) review national health surveys.

## Nigeria

**Leveraging drug vendors **

Jenny Liu et al. (267–275) count drug shops, assess stocks and interview vendors.

## United Republic of Tanzania

### When child survival improves

Almamy Malick Kanté et al. (258–266) study trends in socioeconomic disparities.****

## Global

### Living with HIV

Manjulaa Narasimhan et al. (243–249) survey women about their reproductive health rights.****

### Taxing the global cigarette market

Mark Goodchild et al. (250–257) model the impact of raising tobacco taxes.

### Linking evidence and policy

Abdul Ghaffar et al. (306–308) discuss a relative lack of health policy research. 

